# Noninvasive Positive-Pressure Ventilation for Preeclampsia-Induced Pulmonary Edema: 3 Case Reports and a Literature Review

**DOI:** 10.1155/2018/7274597

**Published:** 2018-08-15

**Authors:** Kohei Hamada, Yoshitsugu Chigusa, Eiji Kondoh, Yusuke Ueda, Shunsuke Kawahara, Haruta Mogami, Akihito Horie, Tsukasa Baba, Masaki Mandai

**Affiliations:** Department of Gynecology and Obstetrics, Kyoto University, 54 Shogoin Kawahara-cho, Sakyo-ku, Kyoto 606-8507, Japan

## Abstract

Pulmonary edema caused by severe preeclampsia can be an indication for pregnancy termination. We aimed to investigate whether noninvasive positive-pressure ventilation (NPPV) was useful for preeclampsia-induced pulmonary edema. Three cases of preeclampsia-induced pulmonary edema managed with NPPV in our institute were reviewed retrospectively. A literature review was conducted regarding NPPV usage during pregnancy. NPPV was initiated at 30, 20, and 24 weeks of gestation in the 3 cases. In all cases, NPPV slowed the progression of pulmonary edema and succeeded in delaying pregnancy termination by 17 days on average. Maternal outcomes were positive, and no intubation was required. Between 1994 and 2017, there were 11 articles describing 12 cases in which NPPV was applied for pulmonary edema during pregnancy. However, there has been no case of NPPV management of preeclampsia-induced pulmonary edema thus far. Maternal and fetal outcomes were positive in these 12 cases. NPPV may contribute to prolonging pregnancy in patients with poor oxygenation due to preeclampsia-induced pulmonary edema. However, patients should be closely monitored, and the decision to intubate or terminate the pregnancy should be made without delay when the maternal or fetal condition worsens.

## 1. Introduction

Preeclampsia, which complicates 3-5% of pregnancies, is still a leading cause of maternal and neonatal mortality. Currently, the only definitive remedy is to deliver the baby and the placenta [1]. Pulmonary edema is a life-threatening complication of preeclampsia, and once it occurs, pregnancy termination is indicated. Considering the mortality and morbidity of neonates, however, obstetricians are tasked with the critical decision of whether to perform an extreme preterm delivery (< 28 weeks of gestation).

Noninvasive positive-pressure ventilation (NPPV) is a form of noninvasive ventilation (NIV), and it refers to the delivery of assisted mechanical ventilation without an invasive endotracheal airway. NPPV employs a full facial mask or nasal cannula that conducts gas from a positive-pressure ventilator into the airways. NPPV has been widely used in patients with chronic respiratory failure, and it has been increasingly applied to patients with various forms of acute respiratory failure [2, 3]. However, few studies have investigated the efficacy of NPPV during pregnancy, and reports describing NPPV management for pulmonary edema due to preeclampsia are scarce.

We herein report three cases of hypoxemic respiratory failure due to pulmonary edema induced by severe preeclampsia that were successfully managed with NPPV. We also review the relevant literature on NPPV usage during pregnancy, especially in the context of preeclampsia-induced pulmonary edema.

## 2. Case 1

A 21-year-old woman pregnant with twins at 29 weeks of gestation was admitted to the previous hospital for preterm labor. After one week of tocolysis with intravenous ritodrine, she developed acute dyspnea and was referred to our hospital. Ritodrine was stopped immediately, and computed tomography of the chest revealed no pulmonary embolus, but bilateral pleural effusion was present. On admission, she also presented with hypertension (152/112 mmHg) and proteinuria (3.8 g/day). She was diagnosed with severe preeclampsia, and magnesium sulfate was initiated, and betamethasone was administered for accelerating fetal lung maturation. After starting magnesium sulfate, her systolic blood pressure did not exceed 140 mmHg, and no further antihypertensive agent was necessary. On day 3 of admission, her SpO_2_ fell to 95% with 5 liters of supplemental oxygen, and NPPV was initiated. After implementation of NPPV, her subjective dyspnea improved, and her SpO_2_ rose to 99% on room air. Pulmonary edema was also ameliorated on her chest X-ray. However, her serum creatinine level was increased to 1.0 mg/dl at 33 weeks of gestation, indicating reduced kidney function. Other symptoms, such as increase in liver enzymes, platelet reduction, and gastrointestinal or neurological symptoms, were not detected. Fetal conditions in utero were favorable. She underwent a cesarean section at 33 weeks and 1 day of gestation due to initiation of labor. The patient delivered healthy male twin infants weighing 1496 g and 1876 g. NPPV was continued intermittently after delivery until she was successfully weaned off of it.

## 3. Case 2

A 36-year-old primigravida at 17 weeks and 4 days of gestation was admitted for hypertension (152/99 mmHg), proteinuria (1.8 g/day), and elevated liver enzymes (AST 75 U/L, ALT 121 U/L). Careful examination revealed no evidence of secondary hypertension or primary renal disease. Furthermore, serum levels of soluble fms-like tyrosine kinase 1 (sFlt1) were very high (8.41 ng/mL) at 18 weeks of gestation. Subsequently, we classified this case as extremely early onset preeclampsia [4]. Nifedipine and magnesium sulfate were administered. Ascites, pleural effusion, and pulmonary edema were detected at 19 weeks of gestation. NPPV was initiated for worsening pleural effusion at 20 weeks of gestation due to desaturation (94% SpO_2_ on room air). After NPPV implementation, the patient's SpO_2_ rose to 99% with 1 liter of supplemental oxygen. Chest X-ray showed no progression of pulmonary edema, although ascites gradually increased, resulting in an emergency cesarean section at 23 weeks and 3 days of gestation due to deteriorating dyspnea, and nonreassuring fetal status, specifically reversed end-diastolic umbilical artery flow and absence of atrial-flow in ductus venosus. A 285 g male infant was delivered. NPPV was discontinued on day 2 after delivery.

## 4. Case 3

A 40-year-old primigravida at 24 weeks of gestation was referred to our hospital for severe hypertension (170/95 mmHg) and proteinuria (8.8 g/day). On admission, she received magnesium sulfate, methyldopa, and nifedipine. On day 2 of admission, she developed respiratory distress with mild desaturation (95% SpO_2_ on room air), and chest X-ray showed bilateral pleural effusion. Blood exam revealed elevation of liver enzymes (AST 133 U/L, ALT 161 U/L), and partial HELLP syndrome was diagnosed. Corticosteroids were administered intravenously, and NPPV was initiated. The patient's SpO_2_ rose to 99%, and pleural effusion did not increase further. However, ascites gradually increased, and her general fatigue became intolerable. As a result, a cesarean section was performed at 25 weeks and 2 days of gestation. Before delivery, the fetal condition in utero was reassuring, in terms of fetal heart rate monitoring and biophysical profile score. A 532 g female baby was delivered. We applied NPPV postoperatively, and she was discharged on day 12 after delivery without any complications.

## 5. Methods of Literature Review

**Figure 1:**
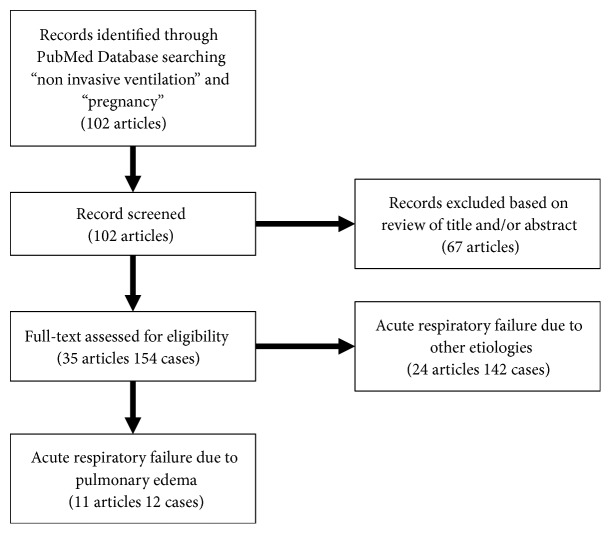
The flow diagram of the trial selection process for this literature review.

A literature review was conducted by searching PubMed for cohort studies or case series using the terms “non-invasive ventilation” AND “pregnancy” between 1994 and 2017. The articles eligible for the study included primary studies that reported clinical data on NPPV usage during pregnancy or parturition. Thirty-five articles including 154 cases were extracted for the final assessment (Figure 1), and 11 of these articles described 12 cases in which NPPV was applied due to pulmonary edema (Table 2).

## 6. Discussion

To the best of our knowledge, this study presents the first cases of pulmonary edema due to preeclampsia that were successfully managed with NPPV during pregnancy. By initiating NPPV before parturition, we succeeded in extending gestation by an average of 17 days (Table 1). This increase in gestational time may lead to better neonatal health outcomes.

Pulmonary edema occurs in approximately 2.9% of patients with preeclampsia [5]. The recent case control study showed that the rate of cesarean delivery was significantly higher among preeclamptic women with pulmonary edema (71.1%) than among those who did not have pulmonary edema (47%). Further, more than 80% of patients with pulmonary edema require mechanical ventilation [6].

In addition, the negative effect of pulmonary edema on preeclampsia may be associated with high maternal mortality, even though this association was not statistically significant [6]. Thus, acute respiratory failure due to pulmonary edema could be a factor influencing the decision for pregnancy termination. For example, the American Congress of Obstetricians and Gynecologists strongly recommends delivery irrespective of gestational age for preeclamptic women whose condition is complicated with pulmonary edema [7]. However, early preterm delivery, especially at less than 28 weeks of gestation, is associated with high neonatal mortality and morbidity. Moreover, neonates born to mothers with early onset preeclampsia have higher mortality, a lower birth weight, and more neonatal complications than gestational age-matched premature neonates born with other etiologies [8]. Therefore, in cases of preeclampsia-induced pulmonary edema early in gestation, we conclude that managing pulmonary edema and hypertension to prolong the gestational period may strongly impact neonatal outcomes.

NPPV comprises continuous positive airway pressure (CPAP) and bilevel positive airway pressure (BiPAP) and has been widely used in patients with respiratory failure. The goal of the use of NPPV is to improve gas exchange, reduce work of breathing, and avoid endotracheal intubation and its related complications. Several studies have shown that NPPV decreases mortality and the need for intubation in patients with chronic obstructive pulmonary disease (COPD) exacerbation or cardiogenic pulmonary edema [3, 9]. A meta-analysis showed that early NPPV application in patients with acute hypoxemic respiratory failure not associated with COPD or cardiogenic pulmonary edema may decrease the need for intubation, the length of time in the ICU, and ICU mortality [10]. However, there are limited data and evidence regarding NPPV during pregnancy thus far. Moreover, according to our literature review, the majority of cases managed by NPPV during pregnancy were severe pneumonia due to the 2009 pandemic H1N1 influenza [11, 12]. We excluded these cases due to the lack of a description of the development of pulmonary edema. As shown in Table 2, only 11 studies involving 12 cases investigated the use of NPPV for pulmonary edema during pregnancy and postpartum period. Among these reports, NPPV was administered in three patients with pulmonary edema caused by preeclampsia, which resulted in positive maternal outcomes [13–15]. Notably, however, in all three cases, NPPV was initiated intraoperatively or postoperatively, and the prolongation of gestational time was not a primary goal. By contrast, four studies including five cases reported that NPPV was implemented before delivery, although the etiology of pulmonary edema was not preeclampsia. Of the five cases, three resulted in full-term delivery [16–18], while two cases of preterm premature rupture of membranes resulted in prolongation of pregnancy for 2 to 3 days [19]. These cases indicate that NPPV management of pulmonary edema during pregnancy may contribute to increasing the gestational period, as long as the maternal and fetal condition are not exacerbated.

The present case series similarly demonstrated that NPPV was effective in delaying the delivery for patients with preeclampsia-induced pulmonary edema. In case 1, pulmonary edema was well controlled, but labor initiation became a decisive factor in the termination of pregnancy. In cases 2 and 3, interestingly, ascites increased gradually, but pleural effusion did not, demonstrating the efficacy of NPPV. In addition, we have encountered a case of early onset preeclampsia complicated by obstructive sleep apnea. Although the Doppler blood flow study revealed absent umbilical arterial endodiastolic flow at 22 weeks of gestation, it dramatically improved after the implementation of NPPV (data not shown). This case might indicate that NPPV improves maternal oxygenation, thereby contributing to improved fetal condition in uterus. Diane et al. also demonstrated that treatment of sleep disordered breathing in preeclamptic women with nasal CPAP improved fetal activity [20].

In our four cases, NPPV was implemented without serious adverse events: no intubation was required, and no NPPV-related complications, such as aspiration, were observed. NPPV should be administered in carefully selected patients during pregnancy and parturition by an experienced clinician with close monitoring. NPPV is contraindicated in patients with impaired consciousness or hemodynamic instability. It should also be avoided in patients who are uncooperative or unable to protect their airway. Decreased esophageal sphincter tone, increased gastric pressure, and delayed gastric emptying due to an enlarged uterus impose a higher risk of aspiration on pregnant women. Furthermore, NPPV was applied not only during pregnancy but also after delivery in the current study. Pulmonary edema can worsen during the postpartum period by returning blood from the uterus to central circulation, leading to low colloid oncotic pressure and increased vascular permeability [21]. Therefore, close monitoring should be continued along with proper fluid volume management and respiratory support, which is necessary in the postpartum period and during pregnancy. In addition, after the initiation of NPPV for preeclampsia-induced pulmonary edema, the timing of pregnancy termination should always be considered. According to our case series, we advocate making the decision to deliver without delay once SpO_2_, pleural effusion, subjective dyspnea, or fetal well-being deteriorates despite NPPV management.

In conclusion, NPPV can be safely administered to pregnant women with hypoxemic respiratory failure due to preeclampsia-induced pulmonary edema. NPPV may contribute to an increase in gestational time by stabilizing the respiratory condition and delaying the timing of delivery. However, patients should be closely monitored, and the decision to intubate or terminate the pregnancy should be made without delay when maternal or fetal condition worsens.

## Figures and Tables

**Table 1:** Summary of the cases.

Case	Age	Gravida/para	Initial indication of NPPV	Comorbidity	Timing of NPPV implementation (weeks of gestation)	Gestational age at delivery (weeks of gestation)	Duration of NPPV management before delivery (days)
1	21	1 / 0	Pulmonary edema	Preeclampsia Twin pregnancy	30 + 4 / 7	33 + 1/ 7	19

2	36	2 / 0	Pulmonary edema	Preeclampsia	20 + 0 / 7	23 + 3 / 7	25

3	42	1 / 0	Pulmonary edema	Preeclampsia partial HELLP syndrome	24 + 3 / 7	25 + 2 / 7	7

NPPV: noninvasive positive pressure ventilation.

**Table 2:** NPPV management of pulmonary edema during pregnancy.

Author	Year	Etiology of pulmonary edema	Timing of NPPV implementation	Duration of NPPV management
Mazlan	2017	Pneumonia	32 weeks of gestation	3 days

Gibbs	2016	TRALI	28 weeks of gestation	24 hours

Fujita	2014	Tocolytic agent	During CS	22 hours after CS

Allred	2013	Pneumonia	30 weeks of gestation	2 days
			28 weeks of gestation	3 days

Suarez	2011	Preeclampsia	After CS	3 days

Frassanito	2011	ARDS due to sepsis	After CS	12 hours

Erdogan	2010	Preeclampsia	The day of CS	24 hours after CS

Bassani	2009	ARDS due to ATRA syndrome	23 weeks of gestation	10 days

Banga	2009	ARDS due to pneumonia	N / A	3-4 days

Perbet	2008	Tocolytic agent	During labor	NA

Terajima	2006	Preeclampsia	During CS	NA

NPPV: noninvasive positive pressure ventilation. TRALI: transfusion-related acute lung injury. ARDS: acute respiratory distress syndrome. ATRA: all-trans retinoic acid. CS: cesarean section.
